# Exploring Factors Associated With Self‐Reported Parental Spanking and Hitting Out of Anger: Cross‐Sectional and Longitudinal Analyses Using National Panel Data

**DOI:** 10.1111/famp.70201

**Published:** 2026-09-30

**Authors:** Christopher J. Mehus, Ana Mireya Díaz‐Howard, Yunqi He, Jaime Ballard, Yvonne Terry‐McElrath, Mona Mittal, Megan E. Patrick

**Affiliations:** ^1^ Department of Family Social Science University of Minnesota St. Paul Minnesota USA; ^2^ Center for Applied Research and Educational Improvement University of Minnesota St. Paul Minnesota USA; ^3^ Institute for Social Research University of Michigan Ann Arbor Michigan USA; ^4^ School of Public Health University of Maryland College Park Maryland USA

**Keywords:** corporal punishment, parenting, spanking

## Abstract

More than one in three US parents use corporal punishment to discipline their children. The objective of this study is to add to a greater understanding of factors associated with self‐reported corporal punishment, based on two survey items: one that asked about use of spanking and one that asked about hitting out of anger. The second objective was to examine the longitudinal relationship between parents' self‐reported use of spanking at age 35 and their self‐reports of hitting out of anger 5 years later. To accomplish these objectives, national US Monitoring the Future Panel Study data were used as follows. Objective 1: data collected in 1993–2020 from parents age 35 with child(ren) ages 2–12 at home. Objective 2: data collected in 1998–2020 from parents age 40 with child(ren) ages 7–17 at home. We conducted multinomial logistic regression to accomplish the objectives. Most factors significantly associated with increased odds of reporting spanking at age 35 differed from the factors associated with increased odds of reporting hitting out of anger at age 35. Reporting spanking at age 35 was associated with greater odds of reporting hitting out of anger at age 40 (aOR = 2.10, 95% CI: 1.71, 2.58). Depressive symptoms (aOR = 1.24, 95% CI: 1.09, 1.40) and disordered alcohol use (aOR = 1.55, 95% CI: 1.14, 2.11) were associated with greater odds of reporting hitting out of anger. Future research should continue to examine whether different types of corporal punishment have different determinants, whether and in what ways corporal punishment may escalate over time, and whether measurement error may impact this research.

## 1 Introduction

More than one in three United States (US) parents report using corporal punishment, such as spanking, to discipline their children (Finkelhor et al. 2019; Mehus and Patrick 2020). Corporal punishment includes a broad range of behaviors, from non‐injurious, open‐handed spanking, to hitting with an implement, to washing a child's mouth out with soap (Sege and Siegel 2018). More severe levels of corporal punishment may be referred to as harsh corporal punishment and may constitute physical abuse. Most academic literature presents evidence that all forms and levels of corporal punishment are harmful. For example, some have called for spanking to be considered an adverse childhood experience (Afifi, Ford, et al. 2017). Major health organizations in the US like the American Academy of Pediatrics (Sege and Siegel 2018) have published statements opposing corporal punishment, based on evidence of harmful effects of corporal punishment on children (Afifi, Ford, et al. 2017; Afifi, Mota, et al. 2017; Avezum et al. 2023; Cuartas 2022; Gershoff and Grogan‐Kaylor 2016; Gershoff et al. 2018; Kang 2022; MacKenzie et al. 2014; Yang and Wang 2026). The negative effects of corporal punishment have been demonstrated across cultures (Cuartas et al. 2025; Gershoff and Grogan‐Kaylor 2016; Gershoff et al. 2012; Pace et al. 2019; Dede Yildirim et al. 2020). Other studies have presented analyses finding that the negative effects of some forms of corporal punishment, such as spanking, are minimal or insignificant and suggest a more nuanced approach to the topic of corporal punishment (Ferguson 2013; Larzelere et al. 2024).

Given the widespread use of corporal punishment in the US, it is important to understand correlates and predictors of corporal punishment use. More specifically, due to the range of behaviors that may constitute corporal punishment and some disagreement about whether levels of corporal punishment can be distinguished, it is important to examine factors related to various types of corporal punishment, including whether more common corporal punishment predicts additional corporal punishment in the future.

### 1.1 Factors Associated With Corporal Punishment

Previous research has suggested several factors that contribute to or influence parenting behaviors, including corporal punishment. Some of these factors have been conceptualized as being internal to the individual, while others—including some demographic factors—may reflect aspects of contextual stress in US‐based research (Belsky 1984; Belsky and Jaffee 2015; Smith 2010).

At an individual level, studies have highlighted relationships between myriad factors and the use of corporal punishment. For example, parental depressive symptoms are associated with use of corporal punishment, including spanking (Callender et al. 2012; Chung et al. 2022; Jansen et al. 2012). Alcohol misuse (e.g., getting drunk) also has been identified as a risk factor for corporal punishment (Afifi et al. 2019; Berger 2005; Wolock and Magura 1996). Other individual factors related to corporal punishment include values and beliefs. For example, conservative views, some religious views, and favorable views toward corporal punishment have been associated with higher rates of corporal punishment (Ellison et al. 1996; Gagné et al. 2007; Giles‐Sims et al. 1995; Straus 2010).

Some demographic characteristics related to corporal punishment may reflect contextual sources of stress. For example, lower income, lower education, being unmarried, and a greater number of people living in the home have been associated with greater use of corporal punishment (Chung et al. 2022; Finkelhor et al. 2019; Scott and Pinderhughes 2019; Ward et al. 2021) Studies of corporal punishment in US samples often report differences in prevalence by race or ethnicity (Finkelhor et al. 2019); rather than an inherent trait, these differences may result from the influence of sociocultural beliefs and norms as well as systems‐wide inequities (e.g., the impact of racism; Scott and Pinderhughes 2019).

However, the relationships between demographic factors and corporal punishment are often complex. For example, related to sex differences, one study found a link between fathers' employment status and mothers' frequency of physical aggression toward children (measured as a sum of frequency of shaking, hitting with an implement, and spanking) but not a dichotomous measure of spanking following misbehavior (Lee and Guterman 2010). In another study, higher income (within a generally low‐income sample) was negatively related to frequency of physical discipline (a measure including spanking, spanking with an implement, and hitting) but positively related to a dichotomous measure of physical maltreatment (a measure including whether discipline resulted in bruises or involvement of authorities; Scott and Pinderhughes 2019).

Although this topic has been well‐studied, additional research can improve our understanding of factors related to corporal punishment. Because of complex or mixed findings, new studies and data can provide additional perspectives to help disentangle the complexities. In addition, studies of corporal punishment have used varied measures of corporal punishment, with some measuring only specific behaviors and others grouping many behaviors. Therefore, it may be the case that determinants differ between various types of corporal punishment, warranting examination of corporal punishment using varied measures.

### 1.2 Escalation of Corporal Punishment

Some research in the US (Zolotor et al. 2008) and internationally (Ma et al. 2022) has drawn attention to low‐severity corporal punishment as a risk factor for harsher corporal punishment or abuse. This body of research primarily includes an examination of the cross‐sectional relationship between spanking (e.g., spanking, hitting, or slapping the child on the bottom with a bare hand) and physical abuse (e.g., beating up the child or hitting or slapping the child on the face or head). In a retrospective study of undergraduates reflecting on childhood experiences, those who reported being spanked at age 10 were almost 60 times more likely to report physical abuse by their parents at age 10 (Fréchette et al. 2015).

Longitudinal, prospective research examining whether common or low‐severity corporal punishment may change or escalate over time is limited. One study of 6‐ to 8‐year‐old children and a mother found that mothers who reported “mild spanking” (spanking with their hand once a month or less) were more likely to report more frequent spanking or spanking with an object the following year (Lansford et al. 2012). For example, of the mothers who reported mild spanking at age 6, 17% reported more frequent spanking or spanking with an object at age 7, compared to 12% of mothers who reported no spanking at age 6.

In related studies, research has also shown a relationship between corporal punishment in 1 year and the same type of corporal punishment in future years (Berlin et al. 2009; Gershoff et al. 2012). Research also shows that corporal punishment generally increases through early childhood (Socolar et al. 2007), as corporal punishment use has long been observed to be greatest among ages 2–4 years (Finkelhor et al. 2019; Straus and Stewart 1999). Cross‐sectional research has shown an association between corporal punishment and maltreatment (Afifi, Ford, et al. 2017).

### 1.3 Gaps and Objectives

Previous research has identified a wide range of individual and demographic factors related to corporal punishment. However, some lack of clarity in this research arises from the various ways in which corporal punishment has been operationalized. In particular, this study adds to previous research with national data by Finkelhor et al. (2019), which focused on only a single measure of corporal punishment (“Thinking of the past year, about how often have you had to spank or slap your child to get him/her to behave”). Furthermore, there is a dearth of longitudinal research examining within‐parent change in reports of corporal punishment over time. There is room to expand on the important findings by Lansford et al. (2012), given the relatively small sample, the inclusion of only mother–child associations, and the lack of examination of other individual or demographic factors that might impact associations. Additional longitudinal studies using national data and longer timespans are needed to expand the limited knowledge base. Whether reports of corporal punishment change within parents over historical time, across different samples, and using different measures of corporal punishment are important considerations for policies and interventions related to corporal punishment.

The purpose of this study is to explore factors associated with self‐reported corporal punishment, using data collected from 1993 to 2020 from a national sample of parents in the US who participated in the Monitoring the Future (MTF) Panel study. Our first research objective is to examine factors associated with two self‐reported corporal punishment items—self‐reported spanking and self‐reported hitting out of anger—at age 35. Our second research objective is to examine the longitudinal relationship between self‐reported corporal punishment by determining whether parents' reported spanking at age 35 is related to their report of hitting out of anger 5 years later at age 40, controlling for key individual‐level and demographic factors.

## 2 Method

### 2.1 Data and Sample

This study used data collected in the US from 28 consecutive 12th grade cohorts for the cross‐sectional age 35 analyses and 23 consecutive 12th grade cohorts for the longitudinal age 40 analyses. Each year since 1976, the MTF study has surveyed a nationally representative sample of 12th grade students in the US. From the 12th grade sample each year, a random sample of 2450 are selected for long‐term follow up in the MTF Panel study (Patrick et al. 2025). The panel participants are surveyed every 2 years until age 29/30 and every 5 years thereafter.

In the present study, we analyzed MTF Panel data provided by respondents at ages 35 and 40 during the years 1993–2020. Overall wave‐to‐wave response rates for these cohorts averaged 89.5% at age 35 and 93.0% at age 40; of all respondents sampled from these cohorts, an average of 48.4% were retained through age 40 (Patrick et al. 2025). Further details on the analytic samples are shown in Tables S1 and S2. Specifically, the analyses involved two different (but overlapping) analytic samples. The age 35 sample involved 28 12th grade cohorts (1976–2003) surveyed across 28 years (1993–2020). Of the 68,398 individuals sampled to participate in the MTF Panel from these cohorts, 32,120 (47.0%) responded at age 35; of those responding, 18,432 (57.4%) had children ages 2–12 at home and were eligible for the current analysis. The age 40 sample involved 23 12th grade cohorts (1976–1998) surveyed across 23 years (1998–2020). Of the 56,133 individuals sampled to participate in the MTF Panel from these cohorts, 27,838 (49.6%) responded at age 35; 25,755 (45.9%) responded at age 40; and 23,055 (41.1%) responded at both ages 35 and 40 (82.8% of those who responded at age 35 also responded at age 40). Of those responding at both ages, 11,460 (49.7%) had children ages 7–17 at home at age 40 and also provided data on children ages 2–12 at home at age 35, making them eligible for the current analysis. MTF surveys do not ask about corporal punishment at ages earlier than 35. To account for attrition, all analyses were conducted with analysis weights (Patrick, Terry‐McElrath, et al. 2023).

Based on weighted age 35 data, most parents in the analytic sample were married or living with a partner (84%) and white (76%). At age 35, parents in the analytic sample reported *M* = 2.17 (SD = 1.09) children aged 2–12 years. The MTF school‐based sampling design is an effective strategy for generating nationally representative samples of 12th grade students. At age 35 follow up, weighting allows for analyses to appropriately represent the original samples (Patrick, Terry‐McElrath, et al. 2023); it is not expected that the sample is representative of the 35‐year‐old US population (e.g., sampling does not include high school dropouts).

### 2.2 Measures

#### 2.2.1 Self‐Reported Corporal Punishment

Self‐reported corporal punishment was captured by measures asking about spanking and hitting out of anger at ages 35 and 40. Regarding spanking, respondents with children were asked, “How often do you spank your child(ren)?” Seven response options included never or almost never, several times a year, about once a month, 2 or 3 times a month, about once a week, several times a week, and every day. We dichotomized these response options to represent 0 = *never/almost never* versus 1 = *more often*. Providing support for the validity of this measure, the dichotomized rates of self‐reported spanking in these data align with rates reported in other studies (Finkelhor et al. 2019; Mehus and Patrick 2020; Straus and Stewart 1999). Regarding hitting out of anger, a similar item asked: “How often do you lose your temper with your child(ren) and hit them or throw things at them?” with response options and coding that mirrored those of the spanking item. These items have remained unchanged through all years of data collection.

At age 35, the two corporal punishment measures were combined and treated as a three‐level variable: (a) no spanking or hitting out of anger (“none”; 52%), (b) spanking but no hitting out of anger (“spanking”; 34%), and (c) hitting or throwing things out of anger with or without spanking (“hitting”; 9%). We chose to combine respondents who reported hitting only with those who reported both hitting and spanking because only approximately one‐third of those who reported hitting out of anger did not report spanking; coding a group of “hitting out of anger without spanking” respondents would result in a problematically low *n* for this group. The three‐level variable was used as the outcome in the analysis for the first research objective and as a predictor in the analysis for the second research objective. At age 40, a dichotomous measure of self‐reported hitting out of anger (with or without self‐reported spanking) was used as the outcome in the analyses for the second research objective. At age 40, a dichotomous outcome (vs. the trichotomy used for age 35 data) was used for three reasons. First, as noted above, the percentage of respondents who report hitting out of anger without any spanking is very low. Second, focusing on hitting at age 40 aligns with our stated research objective of specifically examining change from the more commonly reported item of spanking. Third, spanking becomes less common as children age (Finkelhor et al. 2019); at age 35, parents had at least one child between the ages of 2–12 years but by age 40 may only have children outside of the age range in which spanking is most commonly used.

#### 2.2.2 Individual and Demographic Factors

Individual and demographic factors related to self‐reported corporal punishment included sex, relationship status, conservative values, educational attainment, race/ethnicity, number of children under age 18, depressive affect, and alcohol use. All of these measures were drawn from the age 35 and age 40 surveys (for age 35 and age 40 analyses, respectively), except for race/ethnicity and sex, which were collected at age 18.

##### 2.2.2.1 Sex

Sex was included as a dichotomous variable (male/female), as this is the only variable available across all MTF data collection waves included in the current analysis.

##### 2.2.2.2 Race/Ethnicity

Race/ethnicity was collected in the 12th grade surveys. Because response options have changed slightly over the years and because of small cell sizes, we coded race/ethnicity as white, Black, Hispanic, or other. We include race/ethnicity as a crude proxy for some shared values and experiences related to systematic oppression (Lett et al. 2022).

##### 2.2.2.3 Relationship Status

Relationship status was coded dichotomously as married or living with a partner and “other”.

##### 2.2.2.4 Conservative Beliefs

Whether respondents held conservative beliefs was based on one item that asked, “How would you describe your political beliefs?” We dichotomized this item to compare “very conservative” and “conservative” to other response options. Other response options included “moderate,” “liberal,” and “none of the above”.

##### 2.2.2.5 Education Level

Bachelor's degree denotes the highest level of education completed and was coded as a bachelor's degree or higher versus some college or lower. Highest level of education was used as a proxy for socioeconomic status.

##### 2.2.2.6 Religion

Frequent religious attendance was measured by one question that asked, “How often do you attend religious services?” We dichotomized this item as “once or twice a month” or “about once a week or more” versus “never” or “rarely”.

##### 2.2.2.7 Family Composition

Number of children under 18 included the number of biological, adopted, or step‐children living at home with the respondent (range: 1–8+).

##### 2.2.2.8 Depressive Affect

Depressive affect was a scale (*α* = 0.80) calculated as the mean of four items measuring hopelessness and meaninglessness of life. Example items include: “How much do you agree or disagree with each of the following? …Life often seems meaningless” and “…The future often seems hopeless.” Response options for each item ranged from 1 = *disagree* to 5 = *agree* (possible scale range: 1–5).

##### 2.2.2.9 Alcohol Use

Alcohol use disorder symptoms were coded as a three‐level categorical variable: no recent use, non‐disordered use (i.e., zero or one reported criteria for alcohol use disorder), and disordered use (i.e., meeting at least two criteria for alcohol use disorder). At age 35, all the alcohol use items were asked in reference to the last 5 years. The same time frame was used for age 40 data collected from 1998 to 2016. Because of changes to measures, after 2016, age 40 data pertained to the past 12 months. This measure has been used previously in MTF research (Patrick, Pang, et al. 2023; Terry‐McElrath and Patrick 2025). In this study, non‐disordered alcohol use was used as the reference group.

##### 2.2.2.10 Covariates

Cohort year was also included as a covariate.

### 2.3 Analysis

We used multinomial logistic regression to examine cross‐sectional associations between individual and demographic factors and the odds of the trichotomous outcome of reporting no spanking or hitting, spanking, or hitting at age 35. We used logistic regression to examine longitudinal associations between the age 35 corporal punishment trichotomy and the odds of self‐reported hitting at age 40, controlling for all key factors. In the age 40 analysis, all control variables were drawn from age 40 data, except for race/ethnicity and sex, which were drawn from age 18 data. We used MPlus (v. 8.2; Muthén and Muthén 2017) because of its ability to handle missing data with Full‐Information Maximum Likelihood, which yields unbiased parameter estimates based on available data and allowed us to retain the full analytic samples for analyses (McDonald and Ho 2002) of 18,432 at age 35 (28 cohorts surveyed from 1993 to 2020) and 11,460 at age 40 (23 cohorts surveyed from 1998 to 2020). All analyses were conducted with MTF Panel analysis weights (Patrick, Terry‐McElrath, et al. 2023). The University of Michigan approved the data collection procedures for the MTF Panel Study; the University of Minnesota determined this study did not constitute human subjects research.

## 3 Results

At age 35, 6133 (34.2%) parents reported spanking but not hitting, and 1654 (9.3%) parents reported hitting (including 1150 who reported both and 504 who reported hitting but not spanking). At age 40, 932 (8.6%) of the total sample reported hitting. See Table 1 for descriptive statistics for all variables.

**TABLE 1:** Demographic and behavioral characteristics of parents.

	Age 35			Age 40		
	Number of participants	Weighted percentage (%)	*M* (SD)	Number of participants	Weighted percentage (%)	*M* (SD)
Sex						
Female	11,278	55.8		7109	57.0	
Male	7154	44.2		4351	43.0	
Race						
White	15,582	76.2		9912	78.7	
Black	1050	11.0		584	9.8	
Hispanic	826	6.9		419	6.2	
Asian	249	1.7		130	1.4	
Other	557	4.1		330	3.9	
Married or living with partner						
No	2621	16.0		1690	16.1	
Yes	15,713	84.0		9577	83.9	
Conservative beliefs						
No	12,111	67.0		5381	55.9	
Yes	6114	33.0		4132	44.1	
Obtained bachelor's degree						
No	10,083	57.7		6177	56.3	
Yes	8127	42.3		5179	43.7	
Frequent religious attendance						
No	9061	49.3		5223	45.6	
Yes	9224	50.7		6153	54.4	
Alcohol use						
No recent use	1953	11.8		1357	13.6	
Non‐disordered use	11,315	63.2		7240	66.4	
Disordered use	4645	24.9		2366	20.1	
Number of children			2.17 (1.09)			2.33 (1.18)
Depressive			1.57 (0.78)			1.57 (0.70)
Corporal punishment (age 35)						
No spanking or hitting	9879	52.4				
Spanking (no hitting)	6133	34.2				
Hitting out of anger	1654	9.3				
Hitting out of anger (age 40)						
No				10,220	91.4	
Yes				932	8.6	

### 3.1 Factors Associated With Self‐Reported Corporal Punishment at Age 35

Results of the age 35 multinomial logistic regression are shown in Table 2. Demographic characteristics and other factors that notably differed in their relation to self‐reported spanking versus self‐reported hitting included sex, race/ethnicity, being married, conservative values, education, alcohol use problems, and depressive affect.

**TABLE 2:** Cross‐sectional multinomial logistic regression predicting spanking and hitting at age 35 (*N* = 18,432).

Variables	Spanking (without hitting)		Hitting out of anger (with or without spanking)	
	aOR	95% CI	aOR	95% CI
Sex				
Female	Ref.		Ref.	
Male	**1.20**	1.11, 1.30	**0.84**	0.73, 0.96
Married or living with partner				
No	Ref.		Ref.	
Yes	**1.54**	1.35, 1.75	1.09	0.92, 1.31
Conservative beliefs				
No	Ref.		Ref.	
Yes	**1.66**	1.53, 1.80	0.91	0.79, 1.06
Obtained bachelor's degree				
No	Ref.		Ref.	
Yes	**0.89**	0.82, 0.96	0.93	0.81, 1.07
Frequent religious attendance				
No	Ref.		Ref.	
Yes	**1.69**	1.56, 1.84	**1.51**	1.32, 1.74
Race				
White	Ref.		Ref.	
Black	**2.13**	1.80, 2.51	**2.77**	2.21, 3.23
Hispanic	1.00	0.83, 1.22	**2.56**	2.02, 3.23
Asian	**0.69**	0.48, 0.98	**1.78**	1.14, 2.78
Other	0.90	0.71, 1.15	1.13	0.80, 1.59
Alcohol use				
Non‐disordered use	Ref.		Ref.	
No recent use	0.99	0.87, 1.12	**1.28**	1.03, 1.60
Disordered use	0.97	0.84, 1.11	**1.56**	1.22, 1.98
Number of children	**1.15**	1.10, 1.20	**1.26**	1.18, 1.34
Depressive symptoms	1.04	0.98, 1.11	**1.51**	1.38, 1.65
Cohort year	**0.99**	0.98, 0.99	**0.97**	0.96, 0.98

*Note:* Referent group = none (no spanking or hitting). aORs indicated to be significant by the 95% CI are bolded.

Being male (vs. female) was associated with greater odds of reporting spanking (vs. none; aOR = 1.20) but lower odds of reporting hitting out of anger (vs. none; aOR = 0.84).

Measures that differentiated between the odds of reporting spanking (vs. none) but did not differentiate between the odds of self‐reported hitting (vs. none) included marital status, conservative values, and education. Both being married or living with a partner (vs. not) and having conservative beliefs (vs. not) were associated with greater odds of reporting spanking (vs. none; aORs 1.54 and 1.66, respectively). In contrast, having a bachelor's degree (vs. not) was associated with lower odds of reporting spanking (vs. none; aOR = 0.89). Associations between race/ethnicity and self‐reported corporal punishment at age 35 were mixed.

Both alcohol use and depressive symptoms differentiated between self‐reported hitting (vs. none) but did not differentiate between self‐reported spanking (vs. none). Compared to non‐disordered alcohol use, both no recent use and disordered drinking were associated with higher odds of self‐reported hitting (aORs 1.28 and 1.56, respectively). Higher depressive symptoms were also associated with higher odds of self‐reported hitting (aOR = 1.51).

Measures that differentiated between both self‐reported spanking and self‐reported hitting (vs. none) included religious attendance, number of children, and cohort year. Frequent religious attendance, higher numbers of children, and earlier cohort year were associated with greater odds of reporting both spanking and hitting (vs. none).

### 3.2 Relationship Between Corporal Punishment Severity at Age 35 and Age 40 Hitting

Results of the age 40 logistic regression model are shown in Table 3. Most notably, reporting spanking (vs. none) at age 35 was associated with more than twice the odds of self‐reported hitting at age 40 (aOR = 2.10). Reporting hitting (vs. none) at age 35 was associated with more than eight times the odds of also reporting hitting at age 40 (aOR = 8.38). The factors of disordered alcohol use (vs. non‐disordered use; aOR = 1.55), higher depressive symptoms (aOR = 1.24), and a higher number of children (aOR = 1.12) were also associated with greater odds of reporting hitting. Some race/ethnicity categories were also significant in the model. No difference in the odds of age 40 self‐reported hitting was observed for sex, marital status, conservative values, education, religious attendance, or cohort.

**TABLE 3:** Longitudinal logistic regression predicting hitting out of anger (vs. other) at age 40 (*N* = 11,460).

	aOR	95% CI
Sex		
Female	Ref.	
Male	0.87	0.73, 1.03
Married or living with partner		
No	Ref.	
Yes	0.81	0.64, 1.03
Conservative beliefs		
No	Ref.	
Yes	0.94	0.77, 1.15
Obtained bachelor's degree		
No	Ref.	
Yes	1.02	0.85, 1.21
Frequent religious attendance		
No	Ref.	
Yes	1.13	0.94, 1.37
Race		
White	Ref.	
Black	**1.41**	1.04, 1.90
Hispanic	**1.45**	1.02, 2.05
Asian	1.37	0.67, 2.83
Other	0.90	0.53, 1.53
Alcohol use^a^		
Non‐disordered use	Ref.	
No recent use	1.22	0.93, 1.60
Disordered use	**1.55**	1.14, 2.11
Number of children	**1.12**	1.03, 1.21
Depressive symptoms	**1.24**	1.09, 1.40
Corporal punishment at 35		
None (no spanking or hitting)	Ref.	
Spanking	**2.10**	1.71, 2.58
Hitting	**8.38**	6.73, 10.43
Cohort year	0.99	0.98, 1.01

*Note:* aORs indicated to be significant by the 95% CI are bolded.

^a^ As described in Section 2.2, the alcohol use measure at age 40 changed in recent years; therefore, as a sensitivity analysis, this age 40 model was also run using age 35 alcohol use, which yielded no substantive difference in the results.

## 4 Discussion

This study used national US data from the MTF Panel study to explore the parent factors associated with self‐reported spanking, factors associated with self‐reported hitting out of anger, and whether self‐reported spanking at age 35 is related to the odds of self‐reported hitting at age 40.

Our results showed differences between factors that were associated with age 35 self‐reported spanking (vs. none) and those associated with self‐reported hitting (vs. none). Factors that may be related with more traditional values (religiosity, conservative beliefs) were positively associated with spanking, as others have found (Ellison et al. 1996; Gagné et al. 2007; Giles‐Sims et al. 1995; Straus 2010); these factors were not associated with self‐reported hitting, which was a marked difference between the factors associated with the two corporal punishment items. Partially aligning with previous research, disordered alcohol use (Afifi et al. 2019; Berger 2005; Wolock and Magura 1996) and depressive symptoms (Callender et al. 2012; Chung et al. 2022; Jansen et al. 2012) were associated with self‐reported hitting out of anger but not spanking.

The difference in factors associated with the more‐commonly reported behavior of spanking compared to those associated with self‐reported hitting out of anger suggests that the behaviors may have different origins and therefore would have different potential mechanisms for prevention and intervention. It may also be the case that the results are related to variation in perceptions of the terms spanking or hitting, whether or not they capture differences in behavior. Significant predictors of reporting spanking included more demographic measures than were observed for predictors of reporting hitting out of anger. Many predictors of hitting out of anger in our analyses could be conceptualized as being related to mental health and/or stress. A further examination of findings in this study may help illuminate the unique origins of different levels of corporal punishment. For example, nonusers of alcohol make up a small portion of the sample and may have unique characteristics that are associated with an increased risk of hitting; alcohol research has shown that non‐use (vs. occasional alcohol use) is associated with poorer social and health outcomes (Sarich et al. 2024). The opposite effect of sex between spanking and hitting may be related to time with children or some other process.

Of course, any single interpretation of these results is oversimplifying a complex set of behaviors and factors. For example, race/ethnicity was a significant predictor of spanking and hitting out of anger. Research has suggested that greater use of corporal punishment among some minoritized groups is the result of an internalized white‐supremacist belief that corporal punishment is needed to ensure the level of conduct needed for safety in society (Patton et al. 2021). Others have highlighted the ways in which perceived social norms in favor of corporal punishment within one's community can create a positive feedback loop that perpetuates its use (Duong et al. 2022; Fleckman et al. 2019). It may also be the case that some differences in corporal punishment between racial or ethnic groups are a result of taxed psychological resources among some parents in some minoritized groups, resulting from systemic racism.

We also examined predictors of self‐reported hitting out of anger at age 40; predictors included reporting spanking and hitting at age 35. Importantly, self‐reported spanking at age 35 was associated with over two times the odds of self‐reported hitting at age 40 (vs. none). The results from the age 40 analysis may support the idea that spanking is a risk factor for later additional corporal punishment (Lansford et al. 2012; Ma et al. 2022; Zolotor et al. 2008). The present study adds another longitudinal, prospective analysis to the literature, complementing Lansford et al. (2012). Our analysis included national samples of US male and female participants based on parent age (35 years), with children between the ages of 2–12, across the years of 1993–2020, whereas Lansford included mothers of children 6–8 years old; our time lag was 5 years (parent age 35 to age 40), whereas Lansford's was 1 year across 1988–1991. But both studies showed increased risk of later self‐reported additional corporal punishment among parents who reported spanking.

Other predictors of reporting age 40 hitting included reporting hitting at age 35, alcohol use, number of children, depressive symptoms, and race/ethnicity. Reporting hitting at age 35 was associated with over eight times the odds of reporting hitting out of anger at age 40 (vs. none). The findings for alcohol use, number of children, depressive symptoms, and race/ethnicity were notable in that these measures were associated with self‐reported corporal punishment behavior at age 35 but continued to be significantly related at age 40, even when including age 35 reports of corporal punishment (which would theoretically account for all of age 35 associated risk factor variance). Thus, the observed significant associations remaining at age 40 reflect incremental associations above and beyond those associated with these risk factors at age 35. Aligned with previous research, disordered alcohol use (Afifi et al. 2019; Berger 2005; Wolock and Magura 1996) and depressive symptoms (Callender et al. 2012; Chung et al. 2022; Jansen et al. 2012) were also associated with self‐reported hitting at age 40, even when accounting for age 35 self‐reported spanking and hitting.

Interestingly, our study was able to document decreasing odds across historical cohorts in both reporting spanking and hitting at age 35 (vs. none), but no similar change across cohorts in the odds of reporting hitting (vs. none) at age 40, once controlling for age 35 factors. This partially aligns with prior research, which has found decreasing rates of spanking over historical time (Mehus and Patrick 2020) and indications that harsh physical punishment may be decreasing among younger cohorts of parents (Taillieu et al. 2014).

### 4.1 Strengths and Limitations

A key strength of the study is the use of data from large, national samples of US adults in the general population, which increases potential generalizability. The MTF Panel is subject to attrition (as are all longitudinal studies); the use of MTF Panel analysis weights has been shown to successfully retain the sociodemographic distributions of the initial 12th grade samples (Patrick, Terry‐McElrath, et al. 2023). The degree to which attrition may impact estimates of self‐reported corporal punishment among parents of children at the specified ages is unknown, but there may be some degree of underestimation. The analysis examining the self‐reported hitting at age 40 adds a valuable longitudinal perspective to the literature. The uniform modal age of our sample is a strength in that it reduces possible variance related to parental life stage; however, the results of these analyses may be different if examined at other parental ages. The primary limitation of the study is that only two survey items were available to assess corporal punishment, precluding examination of a wider range of behaviors. The precision of the items is limited by their brevity (including not clearly specifying a time context); each parent or different cultural groups may define spanking differently and there may be some overlap in the behaviors captured by each measure. However, the findings appear to validate that these items capture different constructs or, at least, are perceived as different by parents. In addition, we do not know the exact ages of the children being spanked or hit.

## 5 Conclusion

These findings offer a range of directions for future research. If the survey items in these data successfully captured different types of corporal punishment, then this study suggests that different types of corporal punishment may have different origins. Future research should continue to examine whether this is the case, as it would have implications for intervention and prevention. On the other hand, as is true in all survey research, it may be the case that different participants understood or perceived survey items in different ways, resulting in measurement error. Future research should continue to examine the questions posed in this study with more robust and comprehensive measures of corporal punishment. Finally, future research should continue to examine factors that may increase the risk of changing or escalating corporal punishment over time, including the role of substance use and mental health among parents. This study highlights a need for national, longitudinal data with robust measures of parenting behaviors, including corporal punishment. The results of this study provide insight into factors that can be further examined in future studies.

## Funding

This study was supported by research grant R03HD107135 from National Institute of Child Health and Human Development to C. J. Mehus. Data collection was supported by research grants R01DA001411 and R01DA016575 from the National Institute on Drug Abuse. The opinions, findings, and conclusions expressed in this manuscript are those of the authors and do not necessarily reflect those of the National Institutes of Health, the National Institute of Child Health and Human Development, or the National Institute on Drug Abuse.

## Conflicts of Interest

The authors declare no conflicts of interest.

## Supporting information


**Table S1:** Sample definition for the current study.
**Table S2:** Sample sizes for the current study.

## Data Availability

The data that support the findings of this study are available from https://monitoringthefuture.org/. Restrictions apply to the availability of these data, which were used under license for this study. Data are available from the author(s) with the permission of https://monitoringthefuture.org/.
