# Supplementary material for: Exploring Factors Associated With Self‐Reported Parental Spanking and Hitting Out of Anger: Cross‐Sectional and Longitudinal Analyses Using National Panel Data
Source: Fam Process. 2026 Sep 30;65(4):e70201. doi: 10.1111/famp.70201 (PMC13626966; doi:10.1111/famp.70201)
Supplement: Supplementary file 1 [file FAMP-65-0-s001.docx]

**Supplemental Materials**

**Table S1**

*Sample Definition for the Current Study*

| **12^th^ Grade Cohort** | **Age 19/20** | | **Age 21/22** | | **Age 23/24** | | **Age 25/26** | | **Age 27/28** | | **Age 29/30** | | **Age 35** | **Age 40** |
| --- | --- | --- | --- | --- | --- | --- | --- | --- | --- | --- | --- | --- | --- | --- |
| **1976** | 1977 | 1978 | 1979 | 1980 | 1981 | 1982 | 1983 | 1984 | 1985 | 1986 | 1987 | 1988 | 1993 | 1998 |
| **1977** | 1978 | 1979 | 1980 | 1981 | 1982 | 1983 | 1984 | 1985 | 1986 | 1987 | 1988 | 1989 | 1994 | 1999 |
| **1978** | 1979 | 1980 | 1981 | 1982 | 1983 | 1984 | 1985 | 1986 | 1987 | 1988 | 1989 | 1990 | 1995 | 2000 |
| **1979** | 1980 | 1981 | 1982 | 1983 | 1984 | 1985 | 1986 | 1987 | 1988 | 1989 | 1990 | 1991 | 1996 | 2001 |
| **1980** | 1981 | 1982 | 1983 | 1984 | 1985 | 1986 | 1987 | 1988 | 1989 | 1990 | 1991 | 1992 | 1997 | 2002 |
| **1981** | 1982 | 1983 | 1984 | 1985 | 1986 | 1987 | 1988 | 1989 | 1990 | 1991 | 1992 | 1993 | 1998 | 2003 |
| **1982** | 1983 | 1984 | 1985 | 1986 | 1987 | 1988 | 1989 | 1990 | 1991 | 1992 | 1993 | 1994 | 1999 | 2004 |
| **1983** | 1984 | 1985 | 1986 | 1987 | 1988 | 1989 | 1990 | 1991 | 1992 | 1993 | 1994 | 1995 | 2000 | 2005 |
| **1984** | 1985 | 1986 | 1987 | 1988 | 1989 | 1990 | 1991 | 1992 | 1993 | 1994 | 1995 | 1996 | 2001 | 2006 |
| **1985** | 1986 | 1987 | 1988 | 1989 | 1990 | 1991 | 1992 | 1993 | 1994 | 1995 | 1996 | 1997 | 2002 | 2007 |
| **1986** | 1987 | 1988 | 1989 | 1990 | 1991 | 1992 | 1993 | 1994 | 1995 | 1996 | 1997 | 1998 | 2003 | 2008 |
| **1987** | 1988 | 1989 | 1990 | 1991 | 1992 | 1993 | 1994 | 1995 | 1996 | 1997 | 1998 | 1999 | 2004 | 2009 |
| **1988** | 1989 | 1990 | 1991 | 1992 | 1993 | 1994 | 1995 | 1996 | 1997 | 1998 | 1999 | 2000 | 2005 | 2010 |
| **1989** | 1990 | 1991 | 1992 | 1993 | 1994 | 1995 | 1996 | 1997 | 1998 | 1999 | 2000 | 2001 | 2006 | 2011 |
| **1990** | 1991 | 1992 | 1993 | 1994 | 1995 | 1996 | 1997 | 1998 | 1999 | 2000 | 2001 | 2002 | 2007 | 2012 |
| **1991** | 1992 | 1993 | 1994 | 1995 | 1996 | 1997 | 1998 | 1999 | 2000 | 2001 | 2002 | 2003 | 2008 | 2013 |
| **1992** | 1993 | 1994 | 1995 | 1996 | 1997 | 1998 | 1999 | 2000 | 2001 | 2002 | 2003 | 2004 | 2009 | 2014 |
| **1993** | 1994 | 1995 | 1996 | 1997 | 1998 | 1999 | 2000 | 2001 | 2002 | 2003 | 2004 | 2005 | 2010 | 2015 |
| **1994** | 1995 | 1996 | 1997 | 1998 | 1999 | 2000 | 2001 | 2002 | 2003 | 2004 | 2005 | 2006 | 2011 | 2016 |
| **1995** | 1996 | 1997 | 1998 | 1999 | 2000 | 2001 | 2002 | 2003 | 2004 | 2005 | 2006 | 2007 | 2012 | 2017 |
| **1996** | 1997 | 1998 | 1999 | 2000 | 2001 | 2002 | 2003 | 2004 | 2005 | 2006 | 2007 | 2008 | 2013 | 2018 |
| **1997** | 1998 | 1999 | 2000 | 2001 | 2002 | 2003 | 2004 | 2005 | 2006 | 2007 | 2008 | 2009 | 2014 | 2019 |
| **1998** | 1999 | 2000 | 2001 | 2002 | 2003 | 2004 | 2005 | 2006 | 2007 | 2008 | 2009 | 2010 | 2015 | 2020 |
| **1999** | 2000 | 2001 | 2002 | 2003 | 2004 | 2005 | 2006 | 2007 | 2008 | 2009 | 2010 | 2011 | 2016 |  |
| **2000** | 2001 | 2002 | 2003 | 2004 | 2005 | 2006 | 2007 | 2008 | 2009 | 2010 | 2011 | 2012 | 2017 |  |
| **2001** | 2002 | 2003 | 2004 | 2005 | 2006 | 2007 | 2008 | 2009 | 2010 | 2011 | 2012 | 2013 | 2018 |  |
| **2002** | 2003 | 2004 | 2005 | 2006 | 2007 | 2008 | 2009 | 2010 | 2011 | 2012 | 2013 | 2014 | 2019 |  |
| **2003** | 2004 | 2005 | 2006 | 2007 | 2008 | 2009 | 2010 | 2011 | 2012 | 2013 | 2014 | 2015 | 2020 |  |

**Table S2**

*Sample Sizes for the Current Study*

|  | **Age 35 Analysis** |
| --- | --- |
| 12^th^ Grade cohorts | 1976-2003 (28 cohorts) |
| Years of data collection | 1993-2020 (28 years) |
| Total sampled for MTF Panel from noted cohorts | 68,398 |
| Responded at age 35 ^a^ | 32,120 (47.0% of 68,398) |
| Have children ages 2-12 at home at age 35 (eligible for Objective 1 analysis) | 18,432 (57.4% of 32,120) |
|  |  |
|  | **Age 40 Analysis** |
| 12^th^ Grade cohorts | 1976-1998 (23 cohorts) |
| Years of data collection | 1998-2020 (23 years) |
| Total sampled for MTF Panel from noted cohorts | 56,133 |
| Responded at age 35 ^a^ | 27,838 (49.6% of 56,133) |
| Responded at age 40 ^a^ | 25,755 (45.9% of 56,133) |
| Responded at both ages 35 and 40 ^a^ | 23,055  (41.1% of 56,133;  82.8% of the 27,838 who respond at age 35) |
| Have children ages 7-17 at home at age 40 and data on children ages 2012 at home at ages 35 (eligible for Objective 2 analysis) | 11,460 (49.7% of 23,055) |

*Note.* ^a^ Percentages responding at the noted ages do not adjust for respondents who were deceased or unable to respond at the noted age, or were determined to be ineligible for MTF Panel participation due to being a foreign exchange student while in 12th grade.
